# mSphere of Influence: The maternal gut–mammary axis and its role in shaping neonatal health

**DOI:** 10.1128/msphere.00556-24

**Published:** 2025-02-26

**Authors:** Stephanie N. Langel

**Affiliations:** 1 Department of Pathology, Center for Global Health and Diseases, Case Western Reserve University School of Medicine, Cleveland, Ohio, USA; University of Michigan, Ann Arbor, Michigan, USA

**Keywords:** maternal immunity, breast milk immunity, infectious disease, intestinal microbiota, passive transfer, IgA, IgA antibody secreting cells, gut-mammary axis

## Abstract

Stephanie Langel works in the field of breast milk immunity and maternal-neonatal health. In this mSphere of Influence article, she reflects on how three pivotal papers—Roux et al. (J Exp Med 146:1311–1322, 1977, https://doi.org/10.1084/jem.146.5.1311), Wilson and Butcher (J Exp Med 200:805–809, 2004, https://doi.org/10.1084/jem.20041069), and Rogier et al. (Proc Natl Acad Sci USA 111:3074–3079, 2014, https://doi.org/10.1073/pnas.1315792111)—made an impact on her by uncovering the critical pathways and mechanisms through which gut-derived IgA-secreting cells migrate to the mammary gland and secrete antibodies against intestinal microbes. These foundational studies shaped her understanding of the gut–mammary axis and continue to inspire her research aimed at advancing maternal and neonatal health through breast milk immunology.

## COMMENTARY

In high school, I worked on a dairy farm, milking cows and caring for calves to save money for college. It was there that I first observed the critical link between milk quality and neonatal health. In dairy cows, maternal immunity, in the form of immunoglobulins, is fully transferred through colostrum—the mother’s first milk—during the first 24 to 48 hours of life (1). I witnessed how low immunoglobulin levels in colostrum compromised a calf’s ability to fight infectious diseases. These experiences sparked my curiosity about breast milk immunity and inspired me to pursue a career exploring the intersection of maternal health and infectious disease.

Maternal immunoglobulins, or antibodies, serve as an infant’s first line of defense against infectious diseases and play a crucial role in shaping the newborn’s microbiome (2). Among the five classes of immunoglobulins—IgG, IgA, IgM, IgD, and IgE—the dominance of IgA or IgG in breast milk depends on the species. In humans, IgA is the dominant antibody in both colostrum and mature milk, surpassing IgG and IgM. While IgG is primarily derived from serum, IgA is mainly produced by local IgA antibody-secreting cells (ASCs) that migrate to the mammary gland (3).

How do IgA ASCs migrate to the mammary gland, and where do they originate? These fundamental questions were central to my Ph.D. research under the mentorship of Dr. Linda Saif. Three pivotal papers profoundly influenced my thinking during those formative years and continue to guide the direction of my independent lab. The first paper, published in 1977 in the *Journal of Experimental Medicine* by Dr. Michael Lamm’s lab, provides foundational insights (4). The study was influenced by earlier findings that neutralizing antibodies against *Escherichia coli* and *Vibrio cholerae* were present in human milk (5, 6). Additionally, immunization experiments in pregnant women suggested that lymphoid cells in gut-associated lymphoid tissue (GALT) migrate to the mammary gland to produce IgA antibodies against intestinal microbes (7). Based on these findings, the authors hypothesized that IgA plasma cells in the mammary gland originate from precursors in the mesenteric lymph nodes (MLN) of the intestine.

To test, Roux et al. conducted elegant experiments in which they labeled mesenteric lymph node (MLN) and peripheral lymph node (PLN) cells from virgin CAF1/J mice with radioactive iodine (^125^I-iododeoxyuridine) and intravenously injected them into virgin, pregnant, lactating, and post-lactating mice. They then quantified the percentage of injected radioactivity per gram of mammary gland tissue to assess the homing of these cells. Their results showed an increase in cell homing to the mammary gland during late pregnancy and lactation compared to virgin and post-lactating mice. The authors also demonstrated that these migrating cells had a propensity to produce IgA and were the same population that regularly migrated to the small intestine, contributing to the abundant IgA ASC pool in the gut lamina propria. These findings highlight the mammary gland’s marked ability, during late pregnancy and lactation, to attract and retain gut-derived IgA ASCs.

How do IgA plasma blasts migrate from the gut to the mammary gland? A pivotal 2004 study in the *Journal of Experimental Medicine* by Dr. Eugene Butcher’s lab provided the answer (8). Emerging research had shown that epithelial chemokines, CCL25 and CCL28, mediate the trafficking of IgA ASCs to mucosal sites, but their role in directing IgA ASCs to the mammary gland was unexplored. CCL28 was a strong candidate due to its receptor, CCR10, being expressed by most IgA ASCs; its ability to attract these cells *in vitro*; and its presence in milk. To test if CCL28 mediates IgA ASC accumulation in the mammary gland and influences IgA levels in milk, Wilson and Butcher treated lactating mice with a function-blocking anti-CCL28 antibody. They found that the treatment nearly completely inhibited IgA ASC accumulation in the mammary gland and significantly reduced IgA secretion into milk. In contrast, the levels of IgG and IgM remained unaffected. Furthermore, pups nursing from anti-CCL28-treated mothers had sevenfold lower levels of IgA in their stool, highlighting CCL28’s critical role in IgA transfer and neonatal gut immunity.

Finally, how does secreted dimeric IgA migrate across mammary epithelial cells to reach the lumen and milk, and what are the consequences if this process is disrupted? These critical questions were addressed by a 2014 study from Dr. Charlotte Kaetzel’s lab, published in *Proceedings of the National Academy of Sciences* (9). It was already known that dimeric IgA binds to the polymeric immunoglobulin receptor (pIgR) on the basolateral surface of epithelial cells for transport into the intestinal lumen as secretory IgA (sIgA). Indeed, overexpression of pIgR in the mammary gland of mice increased sIgA levels in both milk and the stomach contents of suckling mice (10). Building on this, Rogier et al. showed that pIgR-deficient (pIgR−/−) dams had significantly lower sIgA levels in the stomach contents of their pups compared to pIgR+/− dams. The absence of sIgA in pIgR−/− pup intestines led to increased translocation of aerobic bacteria to the mesenteric lymph nodes. One identified microbe, *Ochrobactrum anthropi*, an obligate aerobe, showed similar fecal levels in pups from both pIgR−/− and pIgR+/− dams, suggesting that bacterial translocation was due to the lack of sIgA-mediated barrier protection rather than bacterial overgrowth. These findings align with later human studies showing that sIgA prevents immune dysregulation by restraining systemic IgG responses to commensal microbes (11, 12).

Collectively, these studies, spanning nearly four decades, demonstrate that gut-derived, CCR10-expressing IgA ASCs migrate to the mammary gland. There, they secrete dimeric IgA, which binds to pIgR on the mammary epithelial cells and is transferred into milk as sIgA. More research is needed to identify which IgA ASC clones migrate from the gut to the mammary gland and why. Can we harness this gut–mammary axis to drive clones producing neutralizing antibodies against infectious pathogens or clones that produce anti-commensal antibodies to support the development of the neonatal intestinal microbiome? Are there CCR10+ IgA ASCs from other mucosal sites, such as the nasal passages or lungs, that contribute to the mammary gland’s IgA ASC pool? There is still much to uncover about breast milk immunity to better support both maternal and neonatal health!
